# Electrical Stimulation to Conductive Scaffold Promotes Axonal Regeneration and Remyelination in a Rat Model of Large Nerve Defect

**DOI:** 10.1371/journal.pone.0039526

**Published:** 2012-06-21

**Authors:** Jinghui Huang, Lei Lu, Jianbin Zhang, Xueyu Hu, Yongguang Zhang, Wei Liang, Siyu Wu, Zhuojing Luo

**Affiliations:** 1 Institute of Orthopaedics, Xijing Hospital, The Fourth Military Medical University, Xi’an, China; 2 Department of oral anatomy and physiology, School of Stomatology, The Fourth Military Medical University, Xi’an, China; 3 Department of Occupational and Environmental Health and the Ministry of Education Key Lab of Hazard Assessment and Control in Special Operational Environment, School of Public Health, Fourth Military Medical University, Xi’an, China; 4 Fuzhou General Hospital, Fuzhou, China; 5 Department of Orthopaedics, Daping Hospital, The Third Military Medical University, Chongqing, China; University of Milan-Bicocca, Italy

## Abstract

**Background:**

Electrical stimulation (ES) has been shown to promote nerve regeneration when it was applied to the proximal nerve stump. However, the possible beneficial effect of establishing a local electrical environment between a large nerve defect on nerve regeneration has not been reported in previous studies. The present study attempted to establish a local electrical environment between a large nerve defect, and examined its effect on nerve regeneration and functional recovery.

**Methodology/Findings:**

In the present study, a conductive scaffold was constructed and used to bridge a 15 mm sciatic nerve defect in rats, and intermittent ES (3 V, 20 Hz) was applied to the conductive scaffold to establish an electrical environment at the site of nerve defect. Nerve regeneration and functional recovery were examined after nerve injury repair and ES. We found that axonal regeneration and remyelination of the regenerated axons were significantly enhanced by ES which was applied to conductive scaffold. In addition, both motor and sensory functional recovery was significantly improved and muscle atrophy was partially reversed by ES localized at the conductive scaffold. Further investigations showed that the expression of S-100, BDNF (brain-derived neurotrophic factor), P0 and Par-3 was significantly up-regulated by ES at the conductive scaffold.

**Conclusions/Significance:**

Establishing an electrical environment with ES localized at the conductive scaffold is capable of accelerating nerve regeneration and promoting functional recovery in a 15 mm nerve defect in rats. The findings provide new directions for exploring regenerative approaches to achieve better functional recovery in the treatment of large nerve defect.

## Introduction

Lengthy peripheral nerve defect has been posing a clinical challenge for surgeons over the past decades. Despite the advances in microsurgical techniques, a nerve graft was generally required to provide bridges through which injured axons regenerate into the distal stumps to restore motor and sensory function [1], [2]. However, a lengthy of time was generally required for regenerating axons to penetrate through the graft and find the pathways in the distal nerve stump to reach their target organs. During this period of time, the capacities of axotomized neurons to regenerate and of denervated Schwann cells in the distal nerve stump to support regenerating axons declined over time [3], [4], [5]. When the regenerating axons entered the distal nerve stump after a protracted period of time at the site of graft, adequate trophic support and guiding cues for regenerating axons may not be provided by the deteriorated regenerative environment in the distal nerve stump [3], [4], [5]. Consequently, many of the regenerating axons might fail to reach their target organs and functional recovery might be compromised. Therefore, accelerating the rate of axonal regeneration and shortening the time required for axons to cross the graft may help to improve the motor functional recovery in the treatment of large nerve defect.

Electrical signal is an attractive guiding cue for promoting axonal regeneration in nerve injury repair. Although the beneficial effect of ES on nerve regeneration has been widely reported in rat models of crush injury [6], transected injury [7], and short femoral nerve defects [8], [9], the application of ES in the treatment of large nerve defects has been rare. Different from other types of nerve injuries, a large nerve defect generally requires a nerve graft to bridge the two nerve stumps. The graft provides bridges through which regenerating axons regenerate into the distal nerve stump to restore motor function. Thus far, most of the studies on the repair of large nerve defects have been focusing on optimizing the microstructures of nerve scaffold, or introducing neurotrophic factors or seed cells to scaffolds to provide neurotrophic or contact cues for nerve regeneration [1], [2], [10]. Studies on introducing electrical cues at the local site of scaffold to establish an electrical environment between a large nerve defect has not been reported before. Many *in vitro* studies have shown that ES can not only accelerate the speed of neurite outgrowth, but also guide the growth directions of regenerating nerve fibers [11], [12], [13], [14]. All those findings suggest the importance of establishing an electrical environment between a nerve defect by localizing ES at the graft which was used to bridge the two nerve stumps. The local electrical environment might be beneficial for promoting and guiding axonal regeneration, which holds great potential in improving the outcome of nerve defect repair. However, an electrical environment at the site of graft has not been attempted to be established thus far. Therefore, the present study attempted to establish an electrical environment between a large nerve defect, and examined its effect on nerve regeneration and functional recovery.

In the present study, a conductive scaffold was constructed and used to bridge a 15 mm sciatic nerve defect in rats, and one hour ES (3 V, 20 Hz) was applied to the conductive scaffold to establish an electrical environment at the site of nerve defect. The effect of ES, which was localized at conductive scaffold, on axonal regeneration was examined using morphometric analysis and retrograde labeling. Its effect on functional recovery was investigated by electrophysiological study, behavioral study and histological appearance of target muscle.

## Results

### Structural Characteristics and Conductivity of Scaffolds

**Figure 1:**
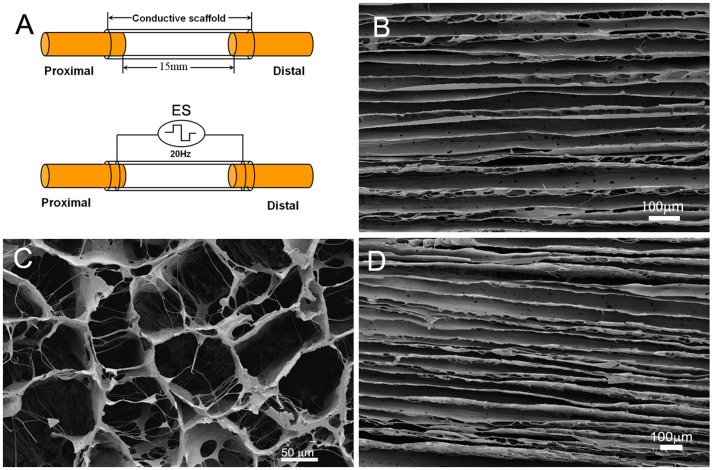
Diagrammatic illustration of combining ES and conductive scaffold in bridging a 15 mm sciatic nerve defect in rats (A), and microstructural characteristics of conductive chitosan/polypyrrole scaffold at longitudinal section (B) and cross section (C), as well as the longitudinal plane of non-conductive chitosan scaffold (D). Scale bar  = 50 µm for (C) and 100 µm for (B, D).

Both conductive polypyrrole/chitosan scaffold and non-conductive chitosan scaffold were fabricated with a longitudinal freezing–dry method. The micro-structure of the conductive polypyrrole/chitosan scaffold was shown in Figure 1. In the longitudinal plane, the polypyrrole/chitosan scaffold showed longitudinally oriented micro-channels (Figure 1B), which was arranged in a honeycomb-like pattern in the cross plane (Figure 1C). The mean diameter of the longitudinal micro-channels was 46.3±7.52 µm (range: 17.1–68.4 µm). The non-conductive chitosan scaffold also exhibited longitudinal micro-channels at its longitudinal section (Figure 1D), with diameter of 45.8±8.41 µm (range: 15.8–72.3 µm), which is in the similar range to that of the polypyrrole/chitosan scaffolds. The conductivity of the polypyrrole/chitosan scaffold was (1.5±0.2)×10^−2 ^S/cm. The conductivity of chitosan scaffold was undetectable.

### ES Localized at the Conductive Scaffold Promotes Axonal Regeneration and Remyelination

**Figure 2:**
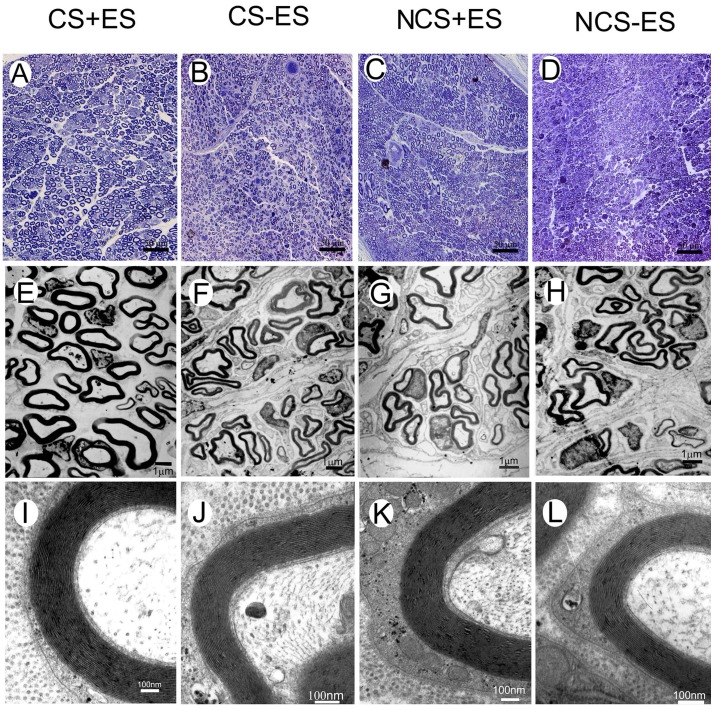
Toluidine blue staining of regenerated axons (A–D), transmission electron micrographs of regenerated axons (E–H) and myelin sheath (I–L) at the distal portion in the CS+ES group (A, E, I), CS−ES group (B, F, J), NCS+ES group (C, G, K) and NCS−ES group (D, H, L) at 12 w post-operatively. Scale bar  = 50 µm for (A), (B), (C) and (D); 1 µm for (E), (F), (G) and (H); 100 nm for (I), (J), (K) and (L).

**Figure 3:**
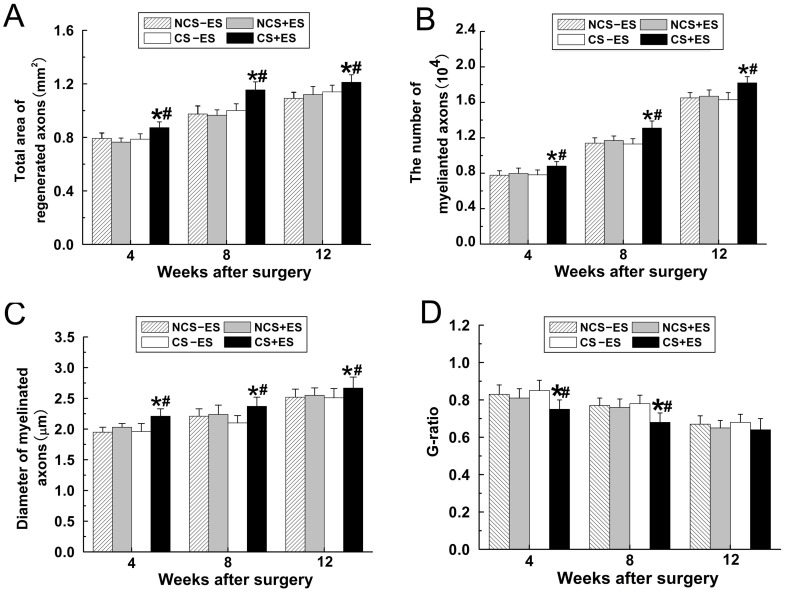
Morphometric analysis of axonal regeneration and remyelination were performed in the CS+ES group, CS−ES group, NCS+ES group and NCS−ES group at the predefined time points. The parameters reflecting axonal regeneration and remyelination includes the cross section area of regenerated nerve (A), quantification of the myelinated axons (B), the diameter of myelinated axon (C), and G-ratio (D) at 4 w, 8 w and 12 w following surgery. All data were expressed as the mean ±standard deviation. *p<0.05 for the comparison with CS−ES group. ^#^ *p*<0.05 for the comparison with NCS+ES group.

**Figure 4:**
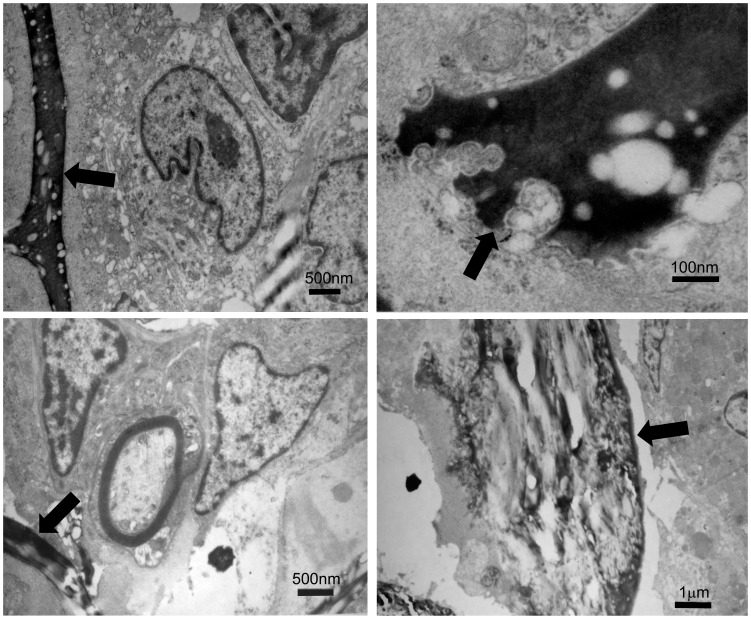
The morphological characteristics of the grafts at 4 w (A, B) and 8 w (C, D) after its *in vivo* implantation. The graft was degraded over time. At 4 w after implantation, the graft showed worm-eroding structures as indicated by black arrows (A, B). At 8 w after implantation, some of the graft degraded into pieces which were indicated by black arrows (C, D).

Conductive scaffold (CS) and non-conductive scaffold (NCS) were used to bridge 15 mm sciatic nerve defects in rats. When one hour ES (3 V, 20 Hz) was applied to the two ends of CS for successive 2 weeks after surgery, the axonal regeneration was significantly enhanced. The total areas of regenerated axons in CS+ES group (rats received CS with ES application, n = 10 at each time point, Figure 2A) were 0.87±0.06 mm^2^ at 4 weeks, 1.16±0.08 mm^2^ at 8 weeks, 1.21±0.07 mm^2^ at 12 weeks, which were significantly higher than those in the CS−ES group (rats received CS with ES application, n = 10 at each time point; Figure 2B, Figure 3A; *p*<0.05, 0.77±0.06 mm^2^ at 4 weeks; *p*<0.05, 1.00±0.07 mm^2^ at 8 weeks; *p*<0.05, 1.12±0.08 mm^2^ at 12 weeks) and in the NCS+ES group (rats received NCS with ES application, n = 10 at each time point; Figure 2C, Figure 3A; *p*<0.05, 0.77±0.05 mm^2^ at 4 weeks; *p*<0.01, 0.97±0.09 mm^2^ at 8 weeks; *p*<0.05, 1.11±0.09 mm^2^ at 12 weeks). The number of myelinated axons in the CS+ES group (Figure 2A; 8815±249 axons at 4 weeks, 13126±413 axons at 8 weeks, 18650±526 axons at 12 weeks) was significantly higher than that in the CS−ES group (Figure 2B, Figure 3B; *p*<0.001, 7821±314 axons at 4 weeks; *p*<0.01, 11335±379 axons at 8 weeks; *p*<0.001, 16345±482 axons at 12 weeks) and NCS+ES group (Figure 2C, Figure 3B; p<0.01, 7984±351 axons at 4 weeks; p<0.01, 11736±368 axons at 8 weeks; p<0.001, 16749±384 axons at 12 weeks). The mean diameter of myelinated axons in the CS+ES group (Figure 2 E; 2.21±0.13 µm at 4 weeks, 2.37±0.12 µm at 8 weeks, 2.67±0.08 µm at 12 weeks) was significantly higher than that in the CS−ES group(Figure 2F, Figure 3C; *p*<0.001, 1.96±0.10 µm at 4 weeks; *p*<0.05, 2.1±0.14 µm at 8 weeks; *p*<0.05, 2.51±0.11 µm at 12 weeks) and NCS+ES group (Figure 2G, Figure 3C; *p*<0.01, 2.03±0.11 µm at 4 weeks; *p*<0.05, 2.24±0.11 µm at 8 weeks; *p*<0.05, 2.55±0.09 µm at 12 weeks). These parameters were in the similar range between the NCS+ES, CS−ES and NCS−ES (NCS without ES application, n = 10 at each time point) groups (*p*>0.05, Fig. 3A–C). This finding suggests that ES localized at the conductive scaffold is able to promote axonal regeneration. In addition, at 4 weeks and 8 weeks after surgery, the remyelination degree of the regenerated axons (G-ratio) in the CS+ES group (Figure 2 I; 0.75±0.05 at 4 weeks; 0.68±0.06 at 8 weeks) was significantly higher than that in the CS−ES group (Figure 2J, Figure 3D; *p*<0.01, 0.84±0.07 at 4 weeks; *p*<0.01, 0.79±0.08 at 8 weeks) and NCS+ES group (Figure 2L; Figure 3D; *p*<0.05, 0.81±0.07 at 4 weeks; *p*<0.05, 0.77±0.07 at 8 weeks). The G-ratio was in the similar range between the CS+ES, CS−ES, NCS+ES and NCS−ES groups at 12 weeks after surgery. These findings indicate that ES is capable of promoting remyelination at the early stage of axonal regeneration. Furthermore, the graft used in the present study is biodegradable. When the graft was implanted, the graft would be degraded over time. At 4 weeks after implantation, the graft showed worm-eroding structures (Figure 4 A, B). At 8 weeks, some of the graft degraded into pieces (Figure 4 C, D). At 12 weeks, it is hard to find residual graft under transmission electron microscopy.

**Figure 5:**
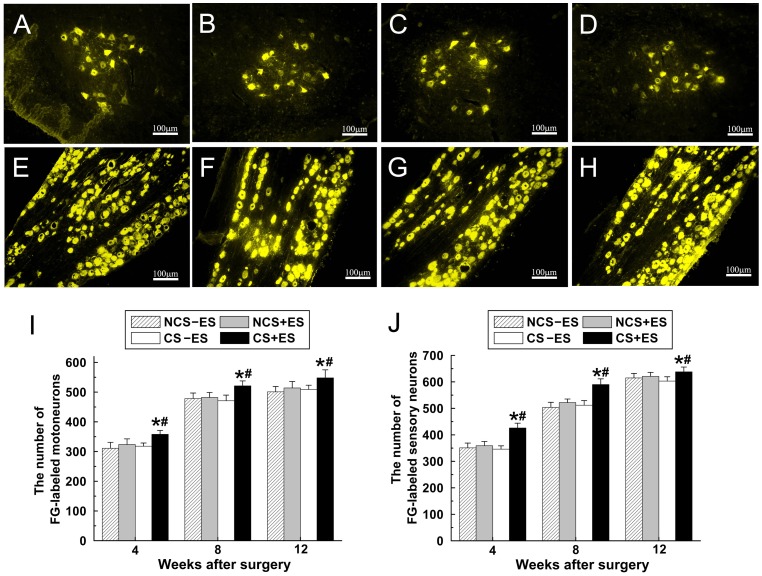
Representative images of FG-positive motoneurons in spinal cord (A–D) and sensory neurons in DRG (E–H) in the CS+ES group (A, E), CS−ES group (B, F), NCS+ES group (C, G) and NCS−ES group (D, H) at 12 w after surgery. The total number of FG-positive motoneurons (I) and sensory neurons (J) which regenerated into distal nerve stump was plotted as a function of time after surgery. All data are expressed as the mean±standard deviation. *p<0.05 for the comparison with CS−ES group. ^#^ *p*<0.05 for the comparison with NCS+ES group.

ES significantly increased the number of Fluoro-Glod (FG) labeled motoneurons in spinal cord and sensory neurons in dorsal root ganglion (DRG, Figure 5A–H). The total number of FG-positive motoneurons in spinal cord in the CS+ES group (Figure 5 I; 358
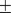
13.1 neurons at 4 week, 521
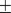
16.8 neurons at 8 week, 548
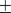
27.1 neurons at 12 week) was significantly higher than that in the CS−ES group (Figure 5 I; *p*<0.001, 318
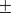
10.4 neurons at 4 week; *p*<0.001, 471
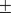
19.1 neurons at 8 week; *p*<0.001, 509
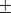
14.3 neurons at 12 week) and NCS+ES group (Figure 5 I; *p*<0.01, 324
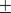
18.7 neurons at 4 week; *p*<0.001, 483
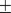
16.9 neurons at 8 week; *p*<0.001, 514
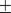
21.8 neurons at 12 week).

In addition, the total number of FG-positive sensory neurons in DRG in the CS+ES group (Figure 5J; 426
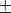
18.1 neurons at 4 week; 590
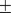
21.5 neurons at 8 week; 638
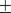
18.2 neurons at 12 week) was significantly higher than that in the CS−ES group (Figure 5J; *p*<0.001, 346
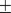
12.6 neurons at 4 week; *p*<0.001, 512
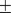
17.4 neurons at 8 week; *p*<0.001, 603
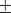
16.3 neurons at 12 week) and NCS+ES group (Figure 5J; *p*<0.001, 359
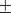
16.1 neurons at 4 week; *p*<0.001, 452
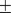
13.4 neurons at 8 week; *p*<0.05, 621
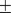
14.8 neurons at 12 week). These findings indicate that nerve fibers, which sprouted from more neurons, successfully regenerated through the conductive scaffold under the effect of ES.

### ES Localized at the Conductive Scaffold Promotes Functional Recovery

**Figure 6:**
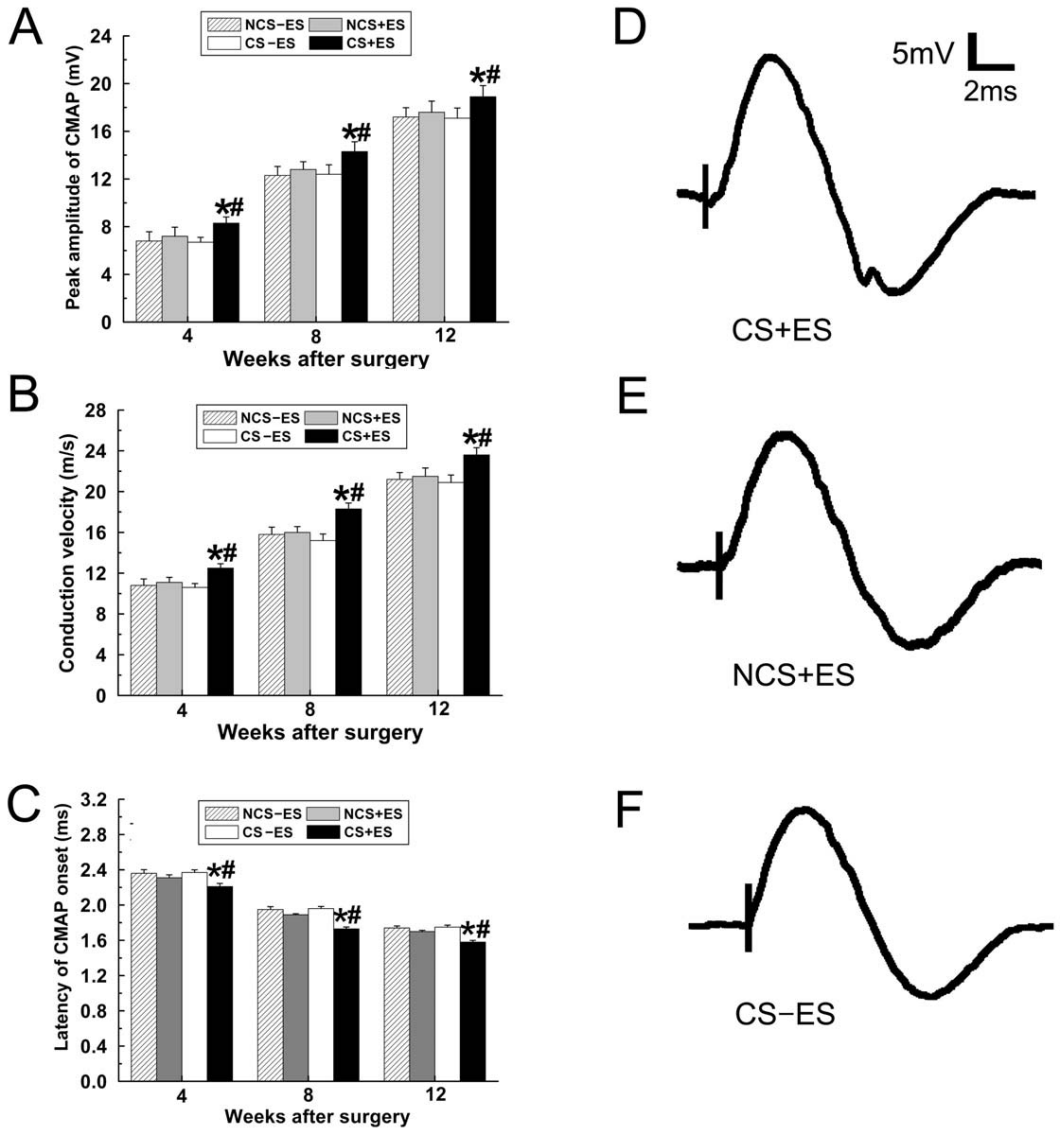
Electrophysiological studies were performed at 4 w, 8 w and 12 w after surgery. Comparisons of amplitude of CMAP (A), NCV (B) and latency onset of CMAP (C) were performed. The Representative recordings from the CS+ES group (D), NCS+ES group (E) and CS−ES group (F) were shown. All data were expressed as the mean±standard deviation. *p<0.05 for the comparison with CS−ES group. ^#^ *p*<0.05 for the comparison with NCS+ES group.

Electrophysiological study was performed to investigate the effect of ES localized at the conductive scaffold on motor functional recovery. As shown in Figure 6, the peak amplitude of the compound muscle action potential (CMAP) in the CS+ES group (Figure 6A; 8.3
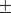
0.52 mV at 4 week, 14.2
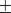
0.83 mV at 8 week, 18.9
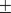
0.93 mV at 12 week) was significantly higher than that in the NCS+ES group (Figure 6A; *p*<0.001, 6.63
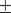
0.41 mV at 4 week; *p*<0.05, 12.4
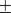
0.79 mV at 8 week; *p*<0.05, 17.1
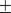
0.85 mV at 12 week) and the CS−ES group (Figure 6A; *p*<0.05, 7.2
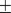
0.76 mV at 4 week; *p*<0.05, 12.8 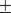
0.65 mV at 8 week; *p*<0.05, 17.9
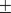
0.93 mV at 12 week). The nerve conducting velocity (NCV) in the CS+ES group (Figure 6B; 12.5
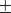
0.43 m/s at 4 week, 18.3
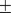
0.58 m/s at 8 week, 23.6
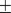
0.71 m/s at 12 week) was significantly higher than that in the NCS+ES group (Figure 6B; *p*<0.001, 10.6
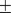
0.39 m/s at 4 week; *p*<0.05, 15.2
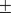
0.65 m/s at 8 week; *p*<0.001, 20.9
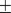
0.73 m/s at 12 week) and the CS−ES group (Figure 6B; *p*<0.01, 11.1
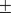
0.49 m/s at 4 week; *p*<0.05, 16.1
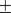
0.57 m/s at 8 week; *p*<0.05, 21.5
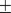
0.82 m/s at 12 week). The CMAP latency of onset in the CS+ES group (Figure 6C; 2.21
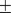
0.04 ms at 4 week, 1.73
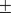
0.02 ms at 8 week, 1.58
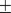
0.02 ms at 12 week) was significantly higher than that in the NCS+ES group (Figure 6C; *p*<0.001, 2.37
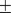
0.03 ms at 4 week; *p*<0.001, 1.96
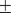
0.02 ms at 8 week; *p*<0.01, 1.75
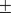
0.02 ms at 12 week) and the CS−ES group (Figure 6C; *p*<0.05, 2.31
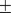
0.03 ms at 4 week; *p*<0.01, 1.89
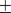
0.01 ms at 8 week; *p*<0.05, 1.70
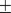
0.01 ms at 12 week). In addition, these three parameters in the NCS+ES group were in the similar range to those in the CS−ES group and NCS−ES group (*p*>0.05, Figure 6A, B, C). The representative electrophysiological image of the CS+ES group, NCS+ES group and CS−ES group were shown in Figure 6 D, E, F). The electrophysiological findings suggest that localized application of ES at the conductive scaffold promotes functional recovery in a 15 mm nerve gap in rats.

**Figure 7:**
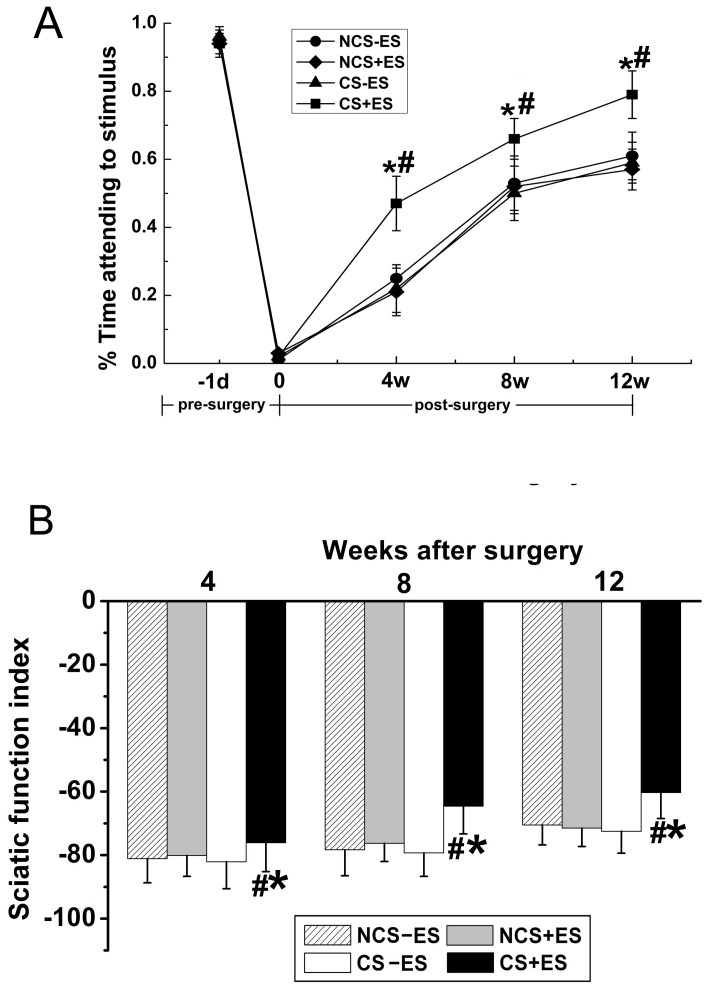
The sticky tape test data (A) and sciatic function index (SFI, B) in all groups at 4 w, 8 w and 12 w after surgery. All data were expressed as the mean±standard deviation. *p<0.05 for the comparison with CS−ES group. ^#^ *p*<0.05 for the comparison with NCS+ES group.

The modified sticky tape test was performed to assess the recovery of somatosensory function after repair. The ratio of the time that the rat spent on attending to the sleeve to the total observation time (30 s) was calculated. We found that the ratio was similar between groups before surgery with 0.95
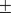
0.04 in the CS+ES group, with 0.93
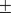
0.03 in the CS−ES group, with 0.91
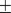
0.04 in the NCS+ES group and with 0.94
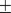
0.05 in the NCS−ES group. After surgery and repair, The ratio in the CS+ES group (Figure 7A, 0.47
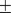
0.08 at 4 week, 0.66
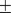
0.07 at 8 week, 0.79
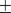
0.09 at 12 w) was significantly higher than that in the CS−ES group (Figure 7A; *p*<0.05, 0.22
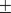
0.08 at 4 week, *p*<0.05, 0.51
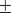
0.09 at 8 week, *p*<0.05, 0.59
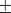
0.08 at 12 week) and NCS+ES group (Figure 7A; *p*<0.05, 0.21
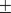
0.07 at 4 week, *p*<0.05, 0.52
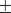
0.08 at 8 week, *p*<0.05, 0.57
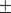
0.06 at 12 week), suggesting that a better sensory recovery was achieved in the CS+ES group.

Sciatic functional index (SFI) was recorded to further examine the effect of ES on motor functional recovery from a behavioral aspect. As shown in Figure 7B, an increase was observed in SFI values in all groups from 4 w to 12 w after surgery (Figure 7B), indicating a spontaneous motor functional recovery after implantation of scaffolds. Further analysis showed that the SFI values in the CS+ES group (Figure 7B, −76.1
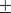
3.23 at 4 week, −64.6
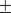
4.18 at 8 week, −60.3
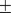
3.25 at 12 week) were significantly higher than that in the CS−ES group (Figure 7B; *p*<0.05, −82.5
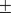
4.74 at 4 week, *p*<0.05, −79.3
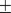
3.90 at 8 week, *p*<0.05, −72.5
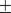
5.66 at 12 week) and NCS+ES group (Figure 7B; *p*<0.05, −80.5
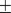
5.57 at 4 week, *p*<0.05, −76.3
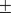
3.49 at 8 week, *p*<0.05, −71.5
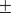
3.62 at 12 week), confirming a better motor functional recovery in the CS+ES group. The SFI values were in the similar range between the CS−ES, NCS−ES and NCS+ES groups (*p*>0.05, Figure 7B).

**Figure 8:**
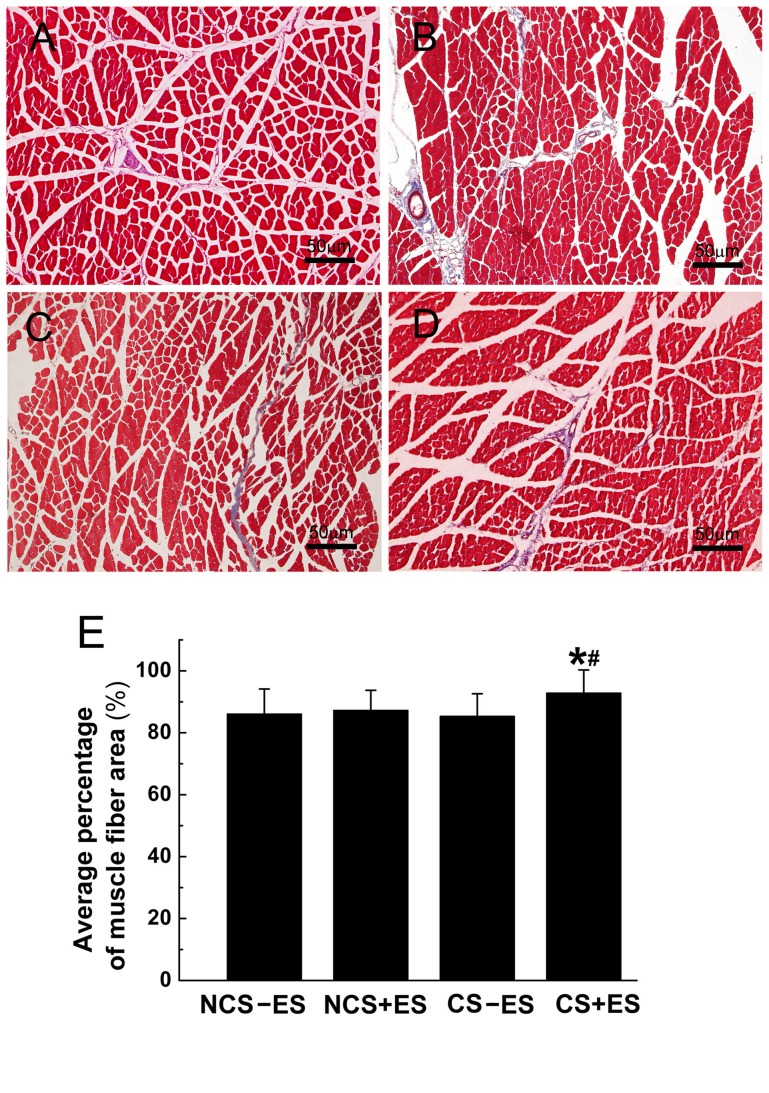
Representative images of transverse sectioned gastrocnemius muscle following Masson trichrome staining for the CS+ES group (A), CS−ES group (B), NCS+ES group (C) and NCS−ES group (D) at 12 w post-operatively. All data were expressed as the mean±standard deviation. *p<0.05 for the comparison with CS−ES group. ^#^ *p*<0.05 for the comparison with NCS+ES group.

The histological appearance of gastrocnemius muscles in each group was assessed by Masson trichrome staining at 12 w after surgery. As shown in Figure 8, the extent of atrophy in the gastrocnemius muscles was lighter in the CS+ES group than that in the CS−ES, NCS+ES and NCS−ES groups (Figure 8A–D). The average percentage of muscle fiber area in the CS+ES group (Figure 8E, 94.4
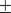
8.7%) was significantly higher than that in the CS−ES group (Figure 8E, *p*<0.05, 85.3
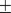
7.5%) and NCS+ES group (Figure 8E, *p*<0.05, 87.6
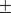
6.9%). The percentage of muscle fiber was not different among the CS−ES group, NCS−ES group and NCS+ES group (*p*>0.05, Figure 8E).

### ES Enhanced the Expression of Regeneration-associated Genes

**Figure 9:**
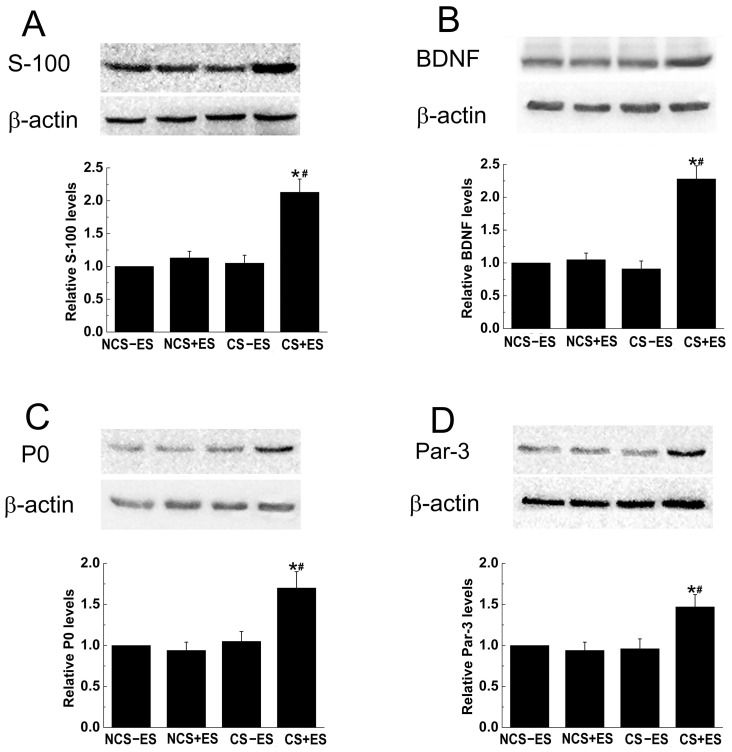
Protein levels of S-100 (A), BDNF (B), P0 (C) and Par-3 (D) at the site of conductive scaffold at 2 weeks after surgery. Each test was repeated three times. The ratio of protein levels from the NCS+ES group, CS−ES group and CS+ES group to that in the NCS−ES group are shown (A, B, C and D). *p<0.05 for the comparison with CS−ES group. ^#^ *p*<0.05 for the comparison with NCS+ES group.

The expression of S-100, BDNF, P0 and Par-3 in each group was examined at 3 weeks after surgery. As shown in Figure 9, The protein levels of S-100, BDNF, P0 and Par-3 was significantly up-regulated in the CS+ES group compared to those in the CS−ES group, NCS+ES group and NCS−ES group (*p*<0.05, Figure 9 A, B, C, D). The Protein levels of S-100 (Figure 9A), BDNF (Figure 9B), P0 (Figure 9C) and Par-3 (Figure 9D) in CS+ES group were 2.02 fold, 2.31 fold, 1.73 fold and 1.56 fold compared to that in CS−ES group, and were 1.88 fold, 2.17 fold, 1.65 fold and 1.53 fold compared to that in NCS+ES group, respectively.

## Discussion

The present study attempted to establish an electrical environment between a nerve defect, and examined its effect on nerve regeneration and functional recovery in a rat model of 15 mm nerve defect. We found that establishment of an electrical environment with ES applied to conductive scaffold was capable of promoting axonal regeneration and remyelination. In addition, ES localized at the conductive scaffold was able to improve functional recovery and muscle atrophy after nerve injury repair. Further investigations showed that the protein levels of S-100, BDNF, P0 and Par-3 were significantly up-regulated by localized ES at the conductive scaffold at 3 weeks after surgical operation. All these findings indicate that establishing an electrical environment with ES localized at the conductive scaffold is capable of accelerating nerve regeneration and promoting functional recovery in a 15 mm nerve defect in rats.

ES localized at conductive scaffold was capable of promoting axonal regeneration in the present study, which was evidenced by the observations that more myelinated axons and larger mean diameter of myelinated axons were observed in rats in which an electrical environment was established between the defect. The beneficial effect of ES on axonal regeneration might be associated with the alteration in the turning responses of the nerve growth cone and enhanced growth capability of growing axons by localized ES [11], [15], [16]. It has been shown that ES applied to *Xenopus* neurons was able to result in a marked alteration in the turning responses of the growth cone induced by gradients of attractive or repulsive guidance cues [16]. When ES was applied to cultured neurons, the neurite trended to elongate along the directions of electric field [12], [13]. In addition, it has also been shown that ES is capable of promoting neurite outgrowth in cultured PC-12 cells *via* conductive polymers [11]. These findings indicate that ES can promote not only the ability of path finding of regenerating axons, but also the growth capability of growing axons, which might partially explain the beneficial effect of ES on axonal regeneration in the present study.

The beneficial effect of ES applied to conductive scaffold on axonal regeneration might be partially related to the effect of ES on Schwann cells. It has been well recognized that migration of Schwann cells into the scaffold is the key for successful axonal regeneration through nerve scaffold [17], [18]. Nerve scaffolds generally achieve better axonal regeneration if they are capable of supporting Schwann cell migration [19], [20]. It has been shown that the migration ability of Schwann cell can be enhanced by exogenous electric field [21]. When localized ES was applied to conductive scaffold in the present study, the migration ability of Schwann cells within conductive scaffold might be enhanced by ES, and the Schwann cell migrated into the scaffold may pave the way for axonal regeneration. In addition, a higher protein level of S-100 (a widely used marker for Schwann cells) was found in the CS+ES group in the present study, indicating that more Schwann cells migrate into conductive scaffold to provide contact guiding cues for axon anchorage and elongation, which might be one of the contributing factors to the beneficial effect of ES on axonal regeneration in this study.

Schwann cells regain the ability of synthesizing neurotrophic factors after peripheral nerve injury, which is of great significance for supporting axonal regeneration and facilitating neuronal repair [22], [23]. It has been shown that up-regulation of neurotrophic factors in Schwann cells can promote axonal regeneration after nerve injuries [24]. Thus, great efforts have been devoted to modulation of the expression of neurotrophic factors in Schwann cells. In our previous *in vitro* studies, electric field has been shown to be able to promote expression and secretion of neurotrophic factors in cultured Schwann cells [25], [26]. In the present study, it was found that the expression of BDNF, an important neurotrophic factor, was up-regulated at the site of conductive scaffold, suggesting that localized ES *in vivo* is also capable of up-regulating the expression of BDNF in Schwann cells. The up-regulated BDNF may provide trophic and guiding cues for regenerating axons and might contribute to the positive effect of ES on axonal regeneration.

Localized ES to conductive scaffold increased the extent of remyelination after nerve injury repair in the present study, which was supported by the findings that thicker myelin sheath and higher G-ratio (axon-fiber diameter) were observed in the CS+ES group. The enhanced remyelination by localized ES might be related to an increased expression of myelination-related genes. In the present study, the protein levels of P0 and Par-3 were significantly increased by ES applied to conductive scaffold. P0 plays an important role in myelin formation and is a key factor for remyelination of regenerating axons [27]. The polarity protein Par-3 localized symmetrically at the sites where Schwann cells contact regenerating axons, and is able to recruit the p75 neurotrophin receptor to the axon-glial junction and form a complex necessary for myelination [28]. The increased expression of P0 and Par-3 by ES indicates that myelin formation was enhanced and cell polarity was increased by localized ES to conductive scaffold. The enhanced remyelination of regenerating axons might be the result of increased myelin formation and facilitated Schwann cell polarization.

Rapid and successful penetrating of regenerating axons through scaffold is of great importance for functional recovery in the treatment of large nerve defect. In the present study, more FG-positive neurons was observed in spinal cord and DRG in the CS+ES group, which indicates that nerve fibers from more neurons successfully penetrate through nerve scaffold. In addition, more myelinated nerve fibers were also observed in the CS+ES group, suggesting that more nerve fibers successfully regenerated through scaffold. These observations indicate that localized ES at the conductive scaffold is capable of facilitating axonal regeneration through conductive scaffold. Further investigations showed that localized ES to conductive scaffold significantly improved motor and sensory functional recovery, as well as muscle atrophy after nerve injury repair in the present study, indicating that successful nerve regeneration through scaffold by ES guarantees functional recovery. Despite the findings that ES localized at the scaffold is capable of promoting motor and sensory functional recovery, we have to realize that the possible beneficial effect of ES on autonomic re-innervations was not examined in the present study. Future studies were needed to identify the effect of ES on autonomic re-innervations.

The findings in the present study are of great therapeutic significance in clinical settings. In comparison to rat, nerve regeneration in humans is a much slower process, and successful penetration of regenerating axons through scaffold is more difficult, which significantly limits functional recovery after a lengthy peripheral nerve defect. When an electrical environment was established between a nerve defect, the localized ES might also promote axonal regeneration and functional recovery in patients with a nerve defect. If regenerated axons which successfully penetrate through scaffold are enhanced by localized ES at conductive scaffold in humans as that described in rats in the present study, it can be expected that the final outcome of lengthy nerve defects would be improved to a considerable extent in humans by establishing an electrical environment between a nerve defect, although this issue remains to be confirmed in larger animals or even humans. In addition, the parameters of ES, including mode (direct current or alternating current), frequency, intensity, and time course are all key factors determining the performance of ES in nerve regeneration. Further studies were needed to identify the optimal parameter of ES which showed best performance in promoting nerve regeneration.

In conclusion, establishment of an electrical environment with ES localized at the conductive scaffold is capable of accelerating nerve regeneration and consequently achieving a better functional recovery in a 15 mm nerve defect in rats. The findings in the present study indicate the importance of establishing an electrical environment at the site of the graft between a nerve defect, and also provide new directions for exploring regenerative approaches to achieve a better functional recovery in the treatment of large nerve defect.

## Materials and Methods

### Fabrication of Conductive Scaffold with Longitudinally Oriented Micro-channels

The conductive polypyrrole/chitosan composite was prepared as described previously [26]. In brief, polypyrrole nanoparticles were synthesized from doubly-distilled pyrrole monomers (98%, Aldrich Chemical Co., Milwaukee, WI, USA) with FeCl_3_ (FeCl_3_: pyrrole, 2.3∶1) as an oxidant. The resulting polypyrrole powder was then suspended in a 0.5% acetic acid aqueous solution. A chitosan solution (3.5% w/v) was made by dissolving chitosan powder (Sigma, USA) in a 1.0% (v/v) acetic acid aqueous solution. polypyrrole was then added into the chitosan solution with vigorous stirring at 65°C for 4 h to get the polypyrrole/chitosan suspension (2.5% w/w polypyrrole).

The preparation of conductive scaffold with longitudinally oriented micro-channels followed the protocol we reported previously [2]. In brief, the polypyrrole/chitosan suspension was degassed under vacuum (50 mTorr) and injected into a hollow, cylindrical copper mold (50.0 mm in length and 2.0 mm in diameter). The mold was vertically placed in a nitrogen canister with the bottom of the mold 20 cm above the liquid nitrogen surface. The mold was then allowed to approach the liquid nitrogen surface with a velocity of 2×10^−5^ m/s. After the chitosan suspensions were completely immersed in liquid nitrogen, they were lyophilized in a freeze-dryer (Alpha 2–4, Chaist, Germany) for 24 h. The polypyrrole/chitosan scaffold were then cross-linked with a 1.0 wt% genipin solution (Wako Pure Chemical Industries, Osaka, Japan) for 24 h, rinsed three times with double-distilled water, dehydrated for 30 min with 95% of ethanol, air-dried for one week, and sterilized with an exposure to 20 kGy ^60^Co radiation prior to animal study.

The preparation and cross-linking of non-conductive chitosan scaffolds with longitudinally oriented pores followed the procedures described as above, during which chitosan solution (1.5% w/v) was used to replace polypyrrole/chitosan. The polypyrrole/chitosan scaffold and chitosan scaffold were sectioned in longitudinal and transverse planes and visualized under scanning electron microscopy (SEM; S-3400N, HITACHI, Tokyo, Japan). The conductivity property of the cross-linked polypyrrole/chitosan and chitosan scaffolds was examined by a four points probe technique at ambient temperature.

### Surgical Procedures and ES

All animal procedures were conducted under a protocol reviewed and approved by the Institutional Ethical Committee of the Fourth Military Medical University (FMMU, No. fmmu-09-5012). Young adult male Sprague-Dawley rats (n = 140, provided by the Laboratory Animal Center of the FMMU, Xi’an, China), weighing from 200 g to 220 g, were anesthetized by intraperitoneal injection of 1.0 wt % sodium pentobarbital solution (40 mg/kg body weight). The left sciatic nerve was exposed by making a skin incision and splitting the underlying muscles in the left lateral thigh. A 15 mm long segment of sciatic nerve was excised and removed, leaving a long defect following retraction of the nerve stumps.

The nerve defects were bridged either with conductive polypyrrole/chitosan scaffolds (n = 70) or with non-conductive chitosan scaffolds (n = 70) with 10/0 nylon sutures of monofilament polyamide under 40× magnification. Half the rats which received conductive scaffolds (n = 35, CS+ES group) and non-conductive scaffolds (n = 35, NCS+ES group) implantation were subjected to ES. In the electrically stimulated rats, two insulated copper wires were bared of insulation for 3–5 mm at their tips, which were to be used as electrodes. One tip of the two wires was twisted to form a loop to get secured into the proximal and distal end of the conductive or non-conductive scaffold before their implantation. When the scaffolds were surgically sutured to the proximal and distal stumps of the sciatic nerve, the other tips of the wires was connected to an ES device (Figure 1A). One hour weak square 0.1 ms electrical pulses (3 V, 20 Hz) were then applied to both the conductive and non-conductive scaffolds. The remaining rats which received conductive (n = 35, CS−ES group) or non-conductive scaffolds (n = 35, NCS−ES group) were sham-stimulated and served as controls. During electrical or sham stimulation, the wound was covered by moistened paper to prevent drying of the underlying tissues. After the first ES, the skin was closed with 4–0 stitches. The tips of the electrode connected to the ES device were secured to the skin using medical adhesive tapes to avoid the rats’ scratching and biting. Then one hour ES (3 V, 20 Hz) was applied to the conductive or non-conductive scaffolds every two days for 8 times for each rat in the CS+ES and NCS+ES groups through the skin tips of the electrodes. ES was applied under anesthesia condition and the tips of the electrodes were re-secured to the skin using medical adhesive taps after stimulation. The rats in the CS−ES group and NCS−ES group were also anaesthetized as that in the CS+ES and NCS+ES groups, but no currents were delivered. Following surgery and stimulation, the animals were retained to their cages and allowed to recovery before starting the functional tests.

### Morphometric Analysis

Four, 8 and 12 weeks after surgery, the regenerated nerves that formed in the place of the grafts were harvested and fixed in 3% glutaraldehyde. The samples (n = 7) were post-fixed in 1% osmium tetroxide in 0.1 M sodium cacodylate buffer (pH 7.3) for 1 h at room temperature, dehydrated in ethanol and embedded in resin. Transverse semi-thin (thickness: 1.0 µm) and ultra-thin (thickness: 50.0 nm) sections were prepared from the distal portion of the graft. The semi-thin sections were stained with a 1% toluidine blue/1% borax solution prepared in distilled water and examined under a light microscope (AH-3, Olympus, Tokyo, Japan). Ultra-thin sections were stained with uranyl acetate and lead citrate, and were examined under a transmission electron microscope (H-600, HITACHI, Tokyo, Japan). Morphometric evaluations were conducted by two independent examiners who were blind to the experimental design. In each group, axonal regeneration was estimated by (1) the total number of myelinated axons per nerve transverse section, (2) the total area of regenerated nerves, (3) the mean diameter of nerve fibers. The degree of remyelination of the regenerated axons was estimated by the axon to fiber diameter ratio (G-ratio).

### FG Retrograde Tracing

Retrograde labeling and counting of back-labeled cells were performed 4, 8 and 12 weeks after nerve injury repair (n = 7 at each time point). Five days before tissue harvesting, the operated sciatic nerve were exposed and 2 µl of 4% FG (Biotium Inc., CO, USA) solution was intraneurally injected into the rat sciatic nerve trunk at a point 2 mm distal to the distal end of the grafts, and the incision was then sutured. The rats were returned to their cages for a period of 5 days to allow the retrograde tracers to travel back to the neuronal cell bodies. Thereafter, the rats were intracardiacally perfused with 4% (w/v) paraformaldehyde in 0.1 M phosphate buffer. The vertebral canal was opened, and the lumber spinal cord was exposed. The L4, L5 and L6 and DRG were then harvested, post-fixed in buffered 4% paraformaldehyde for 4 h, cryoprotected in 30% sucrose overnight at 4°C, and then sectioned on a cryostat. Transverse sections (thickness: 25 µm) were prepared from the spinal cords, and longitudinal sections (thickness:15 µm) were prepared for the DRG. All the sections were mounted on polylysine pre-coated glass slides, which were viewed and photographed under a fluorescent microscope (Olympus BX-60, Tokyo, Japan). The number of FG-labeled spinal cord motoneurons and DRG was counted by two independent investigators who were blind to the experimental groupings.

### Electrophysiological Studies

Four, 8 and 12 weeks after surgery, electrophysiological studies were performed prior to tissue harvesting. After anesthesia was induced, the sciatic nerve was exposed, and the nerve repair site was identified under a surgical microscope. The nerve repair area was insulated from the surrounding muscle with a rubber dam. A bipolar stimulating electrode was placed under the sciatic nerve at a location 10 mm proximal to the graft site. A recording electrode was placed in the gastrocnemius muscle, and the compound muscle action potentials (CMAPs) were recorded. The CMAP peak amplitude, CMAP latency of onset, and nerve conduction velocity (NCV) values were calculated.

### Sciatic Functional Index

Four, 8 and 12 weeks after surgery, walking track analysis was performed on rats (n = 7) to examine the functional recovery as described previously [29]. Pre-operatively, the rats were trained to walk down a wooden track (50×7 cm) into a darkened goal box. The rats’ hind paws were painted with non-toxic finger paint, and any changes in their paw prints that resulted from nerve injury and denervation were recorded. The recordings continued until five measurable footprints were collected. The sciatic functional index (SFI) was calculated as follows:
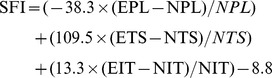
where print length (PL) is the distance from the heel to the top of the third toe, toe spread (TS) is the distance from the first to the fifth toe, and intermediary toe spread (IT) is the distance between the second and the fourth toe. NPL, NTS and NIT represent the PL, IT and TS recorded from the non-operated foot. EPL, ETS, EIT represent the PL, IT and TS recorded from the experimental, operated foot.

### Modified Sticky Tape Test

The modified sticky tape test [30], [31] was performed on the affected hindpaw at 1 d pre-operatively and 4 w, 8 w, and 12 w post-operatively to examine the recovery of sensory sensation (n = 5 in each group). In brief, a paper tape (1.0 cm in width and 3.0 cm in length) was wrapped around the hindpaw in the way that the tape attached to itself and the fingers protrude slightly from the sleeve. The tape sleeve could not be removed once it was wrapped around the hindpaw. The animal was placed in a transparent cage. Then, the responses that the rat attempted to remove the sleeve were recorded, which include pulling at the tape with its mouth and/or brushing the tape with its contralateral paw or forepaws. The rat was observed for 30s. Two timers were started with the first went without stop and the second was turned on only when the rat attempted to remove the tape sleeve. The test was repeated three times per testing day with an interval rest of 2 h and the best two scores were averaged. The ratio of the time that spent on attending to the sleeve to the total time (30 s) was calculated.

### Masson Trichrome Staining for Target Muscles

Twelve weeks after surgery, the gastrocnemius muscle from the operated limbs of rats (n = 7) was harvested from the belly of muscle. The muscle samples were post-fixed with formalin, embedded in paraffin, and processed for Masson trichrome staining. For each sample, photographs were taken from three random fields and analyzed with a Leica software package to measure the transverse section area of the muscle fibers. The percentage of muscle fiber area was calculated as: muscle fiber area/total area [32].

### Expression of Regeneration and Myelination Associated Genes

Three weeks after surgical operation, the regenerated nerves that formed in the place of the grafts were harvested (n = 3), washed with PBS and lysed with lysis buffer containing protease inhibitors (Promega, Madison, WI, USA). The total protein concentration was determined by the BCA assay. Protein extracts were heat denatured at 100°C for 5 min, electrophoretically separated on a 12% SDS-PAGE, and then transferred to a PVDF membrane. The membrane was blocked with 5% non-fat dry milk in TBST buffer (50 mM Tris-HCl, 100 mM NaCl, and 0.1% Tween-20, pH 7.4) and incubated with rabbit anti-rat S-100 antibody (1∶800, Chemicon, USA) and anti-rat BDNF polyclonal antibody (1∶1000, Santa Cruz, USA), anti-rat P0 (1∶1000, Chemicon, USA), mouse anti-rat Par-3 (1∶1000, Chemicon, USA), in 5% non-fat dry milk in TBST buffer at 4°C overnight. The membranes were washed with TBST buffer (3×5 min), and incubated with HRP-conjugated goat anti-rabbit IgG (1∶200, Santa Cruz, USA) or HRP-conjugated goat anti-mouse IgG (1∶200, Santa Cruz, USA) at room temperature for 2 h. The membrane was then washed with PBS and the HRP activity was determined using an ECL kit (USCNLIFE, USA). The image was scanned with a GS 800 Densitometer Scanner (Bio-Rad, Hercules, CA, USA), and the optical density was determined using PDQuest 7.2.0 software (Bio-Rad, Hercules, CA, USA). Rabbit anti-rat β-actin polyclonal antibody (1∶500, Santa Cruz, USA) was used as an internal control.

### Statistical Analysis

All data are expressed as the mean ± standard error of mean (SEM). The data were analyzed using one-way analysis of variance (ANOVA) with the SPSS13.0 software package (SPSS Inc., Chicago, IL, USA). If there was a significant overall difference among groups, Tukey post hoc test was then used to make pair-wise comparisons. Values of *p*<0.05 were considered statistically significant.
